# PAGER 2.0: an update to the pathway, annotated-list and gene-signature electronic repository for Human Network Biology

**DOI:** 10.1093/nar/gkx1040

**Published:** 2017-11-08

**Authors:** Zongliang Yue, Qi Zheng, Michael T Neylon, Minjae Yoo, Jimin Shin, Zhiying Zhao, Aik Choon Tan, Jake Y Chen

**Affiliations:** Informatics Institute, School of Medicine, the University of Alabama at Birmingham, AL 35294, USA; School of Information Science and Technology, Guangdong University of Foreign Studies, Guangzhou, Guangdong 510006, China; Indiana University School of Informatics and Computing, Indiana University-Purdue University Indianapolis, Indianapolis, IN 46202, USA; Division of Medical Oncology, Department of Medicine, University of Colorado Anschutz Medical Campus, Aurora, CO 80045, USA; School of Computer Science and Engineering, Northeastern University, Shenyang 110819, China

## Abstract

Integrative Gene-set, Network and Pathway Analysis (GNPA) is a powerful data analysis approach developed to help interpret high-throughput omics data. In PAGER 1.0, we demonstrated that researchers can gain unbiased and reproducible biological insights with the introduction of PAGs (Pathways, Annotated-lists and Gene-signatures) as the basic data representation elements. In PAGER 2.0, we improve the utility of integrative GNPA by significantly expanding the coverage of PAGs and PAG-to-PAG relationships in the database, defining a new metric to quantify PAG data qualities, and developing new software features to simplify online integrative GNPA. Specifically, we included 84 282 PAGs spanning 24 different data sources that cover human diseases, published gene-expression signatures, drug–gene, miRNA–gene interactions, pathways and tissue-specific gene expressions. We introduced a new normalized Cohesion Coefficient (*nCoCo*) score to assess the biological relevance of genes inside a PAG, and *RP-score* to rank genes and assign gene-specific weights inside a PAG. The companion web interface contains numerous features to help users query and navigate the database content. The database content can be freely downloaded and is compatible with third-party Gene Set Enrichment Analysis tools. We expect PAGER 2.0 to become a major resource in integrative GNPA. PAGER 2.0 is available at http://discovery.informatics.uab.edu/PAGER/.

## INTRODUCTION

In a biological system, multiple genes and proteins regulate in concert to exert specific processes (1–3). To study and decipher these complex biological systems, high-throughput technologies such as microarray, next-generation sequencing and mass spectrometry are routinely used to generate measurements of gene and protein activities at genomic and proteomic scale, respectively. The straightforward analysis is to perform candidate gene analysis to identify statistically significant genes or proteins that are differentially expressed from these ‘omics’ datasets. However, the challenge from the candidate analysis is the interpretation of results. Alternatively, the ‘**G**ene-set, **N**etwork, and **P**athway **A**nalysis’ (**GNPA**) provides an unbiased approach to analyze the ‘omics’ datasets (2). GNPA addresses many of the candidate gene analysis with high reproducibility, model robustness and data interpretability.

Gene Set Enrichment Analysis (GSEA), first introduced to perform a particular variant of GNPA, has revolutionized the data analysis and interpretation of high-throughput omics data (4,5). Accompanying GSEA is the development of Molecular Signature Database (MSigDB) (6–8), which introduced the ‘gene-set’ concepts for GNPA. Inspired by GSEA, many computational tools have been developed over the years to perform GNPA over omics data (2,9). Similarly, various gene signature databases including GeneSigDB (10) and PAGER 1.0 (11) have been developed to incorporate new biological knowledge into GNPA. However, current gene-sets or molecular signatures reside across highly heterogeneous data sources; moreover, these data do not readily capture molecular relationships/context information. This has made GNPA today still a fairly ‘hit-or-miss’ analysis—a laborious process requiring manual evaluations (2,12–15).

To overcome these challenges, we previously developed PAGER, a novel and comprehensive database infrastructure by integrating **PAG**s—a new unified data structure to represent heterogeneous **P**athways (P-type), **A**nnotated-lists (A-type) and **G**ene-signatures (G-type) (11). In PAGER 1.0, we compiled 44 313 genes from five different species including human, 38 663 PAGs, 324 830 gene–gene relationships and 3 174 323 PAG-to-PAG regulatory relationships. We also developed a cohesion measure called **Co**hesion **Co**efficient (*CoCo*) to assist users in assessing the biological relevance within each PAG. However, there are several limitations, including inadequate PAG coverage, lack of functional information and impact of genes in the PAGs, and lack of molecular interaction/regulation details inside PAGs.

In PAGER 2.0, we provide a major update for PAGER to provide substantially expanded PAG data coverage, a new normalized quality score metric called *nCoCo* to assess the biological relevance of genes inside each PAG, and a new user-friendly interface to help users perform integrative GNPA queries. The new PAGER 2.0 contains 84 282 PAGs, 601 164 gene–gene relationships, and 7 538 275 PAG-to-PAG relationships. The PAGs were derived from 24 different data sources that cover, for example, human diseases, published gene expression signatures, known gene lists affected by shared drugs, pathways, shared miRNA–gene interaction targets, tissue-specifically co-expressed genes and all genes sharing common protein functional annotations. The new normalized *CoCo* score (*nCoCo*) employs polynomial regression models to correct for the PAG size bias, which was not considered in the original *CoCo* score. To assist users in prioritizing genes in the PAGs, we integrated the functional gene–gene interaction data from the recently published HAPPI-2 database (16) to generate a gene ranking score (*RP-score*) (17) based on the biological context-specific study. We also provided literature evidence link wherever the gene was found in the context of the PAGs descriptions as reported in PubMed literature. Finally, we improved the web portal for easy navigating, querying, and downloading the PAGER 2.0 database. We intend for PAGER 2.0 to become a major resource for researchers interested in integrative GNPA.

## MATERIALS AND METHODS

### Collections of PAGER 2.0 data sources

PAGER 2.0 consists of 24 data sources, 14 data sources are inherited from the PAGER 1.0 and 10 are new data sources. PAGER 2.0 now covers data sources from diseases (GAD (18), GWAS Catalog (19), PheWAS (20)), gene expression signatures (MSigDB (7), GeneSigDB (10), ImmuneSigDB (21)), drug–gene interactions (PharmGKB (22), DSigDB (23)), pathways (SPIKE (24), WikiPathways (25), Human Pathway Database HPD (26), including HPD–Reactome (27), HPD–BioCarta (28), HPD–PID (29), HPD–KEGG (30)), miRNA–gene interactions (microcosm Targets (31), TargetScan (32), miRTARbase (33)), tissue-specific gene expression (NGS Catalog (34), GTEx (35)), functional annotations (Gene Ontology Annotions (36)), genes (Genome Data) and proteins (Protein Lounge: http://www.proteinlounge.com/Pathway, Pfam (37), Isozyme (38)). The number of PAGs extracted and integrated in PAGER 2.0 is listed in Table 1.

**Table 1.** Statistics of PAGER 2.0 as compared to PAGER 1.0

	PAGER 1.0	PAGER 2.0	Increase ratio
**Genes in PAGs**	44 313	65 774	148%
**Gene–gene relationships**	115 840	601 164	518%
PPI	93 713	579 037	617%
Gene Regulation	22 127	22 127	100%
**PAG**	38 379	84 282	219%
Singleton (*n* = 1)	19 772	27 206	137%
Regular (*n* > 1)	18 607	57 076	306%
with CoCo scores (*n* > 1)	14 701	42 048	286%
with CoCo score ≥ 1	13 856	15 028	108%
**PAG-to-PAG pairs**			
m-type (V1:logPMF > 5 V2:logCDF > 10)	3 101 499	7 418 174	239%
r-type (V1:PMF < 0.05 V2:CDF < 0.05)	72 824	120 101	164%
sPAG to mPAG	7250	28 744	396%
mPAG to mPAG	39 253	83 741	213%
mPAG to sPAG	2479	4613	186%

### Normalized CoCo score calculation

We previously developed *CoCo* score (a Correlation Coefficient derived from the measure of statistically significant coverage of gene–gene functional correlations in gene pairs or gene trios), a quality metric to measure PAGs in PAGER 1.0. However, the limitation of the *CoCo* score is it does not consider the PAG size. Here, we improve the *CoCo* score to a new PAG size-normalized quality metric. The new score—*nCoCo* rescales the original *CoCo* scores based on polynomial regression models to eliminate the PAG size bias (see Supplementary Methods for details). The *nCoCo* score allowed to compare the PAG quality independent of the PAG size shown in Table 2. We have presented an example of the comparison of the *nCoCo* score between PAG WIG001980 ‘Non-homologous end joining’ and PAG WIG001424 ‘Actin Nucleation and Branching’. The result showed that the PAG WIG001424's *CoCo* score is higher than the PAG WIG001980's *CoCo* score due to the size effect (6 versus 101). And in the *nCoCo* score comparison, we were able to explore that quality of PAG WIG001980 is much higher than PAG WIG001424 since the protein–protein interactions (PPIs) of the PAG WIG001980 reached to the upper limit (13 out of 15).

**Table 2.** An example of comparing the PAG quality using *nCoCo* score

PAG Id	Type	PAG name	PAG size	Theoretical PPI	PPI	*CoCo*	*nCoCo*
WIG001980	P	Non-homologous end joining	6	15	13	88	1153
WIG001424	P	Actin Nucleation and Branching	101	5050	612	2094	130

### Gene prioritization within PAGs

We used the *RP-score* gene prioritization algorithm initially reported in (17) to rank the gene prioritization involved with prior knowledge along with PAG information. The concept of a PAG is the gene membership with a certain context. The genes organized in the group are always considered as carrying out some certain function or disease gene signature. The quality of the group is measured by the gene–gene interactions in the group. Our *RP-score* rank utilizes the PAG and the gene–gene interactions to rank the genes based on the gene weight calculated in the PAG and the frequency the genes appear in the PAGs (see Supplementary Methods for details).

### Literature support of the gene members in the PAGs

To support the gene members in the PAGs, we performed biomedical literature mining using the PubMed corpus. We employed GNormPlus (39) to normalize the gene names from literature. For each gene in the PAG, we used the Entrez Programming Utilities (40) to query the gene names together with the PAG name. Using the E-utilities, we retrieved all the PubMed IDs related to both the PAG and the genes. We annotated the sentences containing the gene names in these articles as literature support for the gene members in the PAGs.

### Database and web portal implementation

We used PHP5, Javascript and Codeigniter version 2.1.3 (https://codeigniter.com/) as the web presentation framework and Oracle 12g as the backend database. Real-time calculation of hypergeometric cumulative distribution function (CDF) was implemented with PDL (https://github.com/php-math/PDL), a PHP library for statistics. Cytoscape.js (http://js.cytoscape.org), an open-source graph library, and jQuery were used to visualize gene and PAG networks. D3.js (http://d3js.org/) was used to perform matrix visualizations. DataTables, a plugin for jQuery was used for displaying the tables and enabling download.

## DATABASE CONTENT AND WEB INTERFACE

### Overall statistics of data in PAGER 2.0

The statistics show that PAGER 2.0 has significantly increased the coverage of the PAGs and PAG-to-PAG relationship in Table 1. In brief, PAGER 2.0 contains 65 774 genes in 84 282 PAGs, which contains 601 164 gene–gene relationships and 7 418 174 PAG-to-PAG pairs. The regular PAGs (PAG size > 1) fold change is 3.06. After the *nCoCo* score filtering (*nCoCo* > 1), the PAGs fold change is 2.86. The m-type PAG-to-PAG relationship fold change is 2.39. The m-type PAG-to-PAG relationship fold change is 1.64. Among the r-type PAG-to-PAG relationship, the sPAG-to-mPAG relationship fold change is 3.96, the mPAG-to-mPAG relationship fold change is 2.13, and the mPAG-to-sPAG relationship fold change is 1.86. This represents a substantial improvement in terms of size and coverage of PAGs and PAG-to-PAG relationships from the previous version.

### Statistics of the PAGs in PAGER 2.0

**Figure 1.**
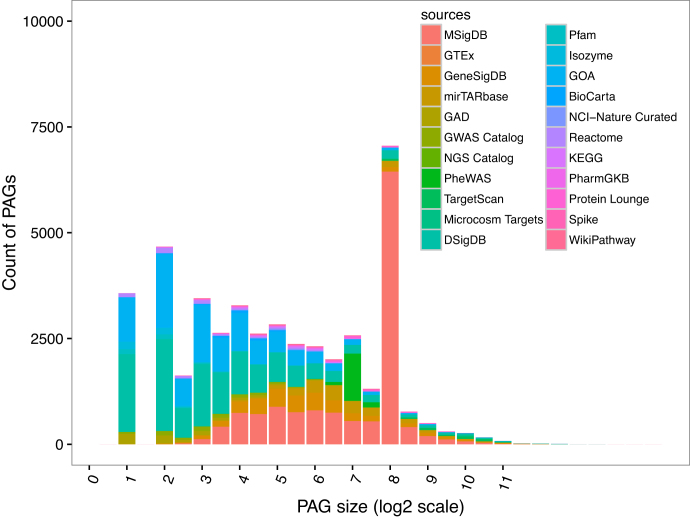
Distribution of the PAG size from 22 data sources. The color indicates the PAGs size distribution from 22 different sources. The MsigDB includes the ImmuSigDB and the Genome Data has not shown since the size is equal to 1.

To evaluate the statistics of PAGs integrated into PAGER 2.0, we investigated the distribution of the PAG sizes in PAGER 2.0. The peak at the PAG size of 200 in the PAG size distribution shown in Figure 1 indicates that there are specific sources that have contributed toward the inflation. Most of the PAGs (44.1%) with size = 200 are contributed by MSigDB. Since MSigDB is a human-curated gene signature database for diseases, the PAG size has a preference. The PAG size distribution is grouped by the PAG type and the derivation method is shown in Supplementary Figure S1, and the identifier of the PAG is shown in Supplementary Table S2.

**Figure 2.**
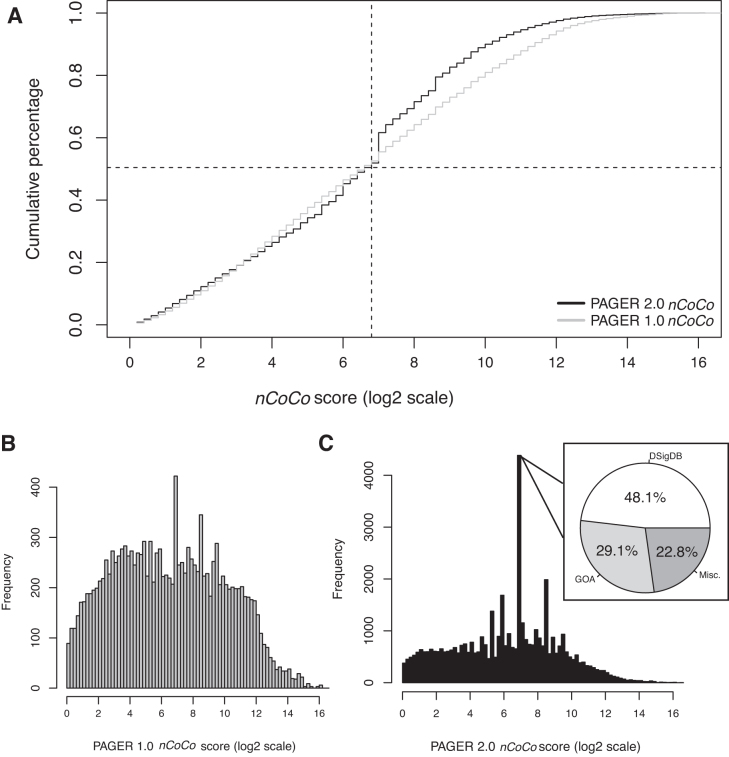
Comparisons of PAGER 2.0 *nCoCo* distribution and PAGER 1.0 *nCoCo* distribution. (**A**) The cumulative percentage of *nCoCo* score in PAGER 1.0 and PAGER 2.0. The gray line is the *nCoCo* score of PAGER 1.0 and the black line is the *nCoCo* score of PAGER 2.0. The dash line indicates the cross point of the *nCoCo* score of PAGER 1.0 and PAGER 2.0 at PAG size = 128 and cumulative percentage = 0.50. (**B**) PAGER 1.0 *nCoCo* distribution. The bin size is in increments of 2^0.2^ to form the range of [2^x^,2^x+0.2^]. x ranges from 0 to 16. (**C**) PAGER-2.0 *nCoCo* distribution. The pie-chart shows the *nCoCo* bin of [2^6.8^,2^7^].

To evaluate the biological relevance of each PAG in PAGER 2.0, we computed the *nCoCo* score for all the PAGs in PAGER 2.0 and compared with PAGER-1.0. The *nCoCo* score calculation distribution is shown in Supplementary Figure S2. In the comparison of the frequency of *nCoCo* score from PAGER 1.0 and PAGER 2.0 in Figure 2, the difference between PAGER 1.0 and PAGER 2.0's *nCoCo* score below the 50% is not significant (0.014 ± 0.010) and similarly not significant when comparing the difference between PAGER 1.0 and PAGER 2.0's *nCoCo* score above the 50% (0.057 ± 0.032). The break point of the frequency change is due to the inflated *nCoCo* score = 128 shown in Figure 2C. The major sources of the *nCoCo* score bin of size ranging from 2^6.8^ to 2^7^ in PAGER 2.0 are from DSigDB (48.1 %) and GO term (29.1%). Since the PAGs of DSigDB and GO term consists of functional biological biomarkers, the quality of the PAG is relatively good as the score is concentrated in the bin of size ranging from 2^6.8^ to 2^7^.

After calculating the *nCoCo* score at the cumulative percentage of 50% (*CP50*), the quality of the 10 new sources in PAGER 2.0 has been compared with the overall quality of PAGER 2.0 in Supplementary Figure S3. The source’s *nCoCo* score at *CP50* indicates the quality of PAGs from the source. A relative larger *nCoCo* score at *CP50* suggests the distribution of *nCoCo* is right-skewed. Therefore, the PAGs from DSigDB, Isozyme, GO term and Pfam above the baseline (*nCoCo* score at *CP50* is in the between of 64 and 128) indicate the gene members in the PAGs from these four sources have relatively high interactions and trios. *nCoCo* score cumulative percentage grouped by type is shown in Supplementary Figure S4.

The Supplementary Figure S5 shows the power-law distributions of the m-type PAG-to-PAG relationship and the r-type PAG-to-PAG relationship. This indicates that the m-type PAG’s regulatory network CDF score and the r-type PAG's regulatory network CDF score are strong metrics to stratify the quality level of the m-type PAG-to-PAG relationship and the r-type PAG-to-PAG relationship.

### PAGER 2.0 web interface and user case examples

Users can query the PAGER 2.0 database via the web portal by using the ‘Basic Search’ or ‘Advanced Search’ options. For the ‘Basic Search’, users can query PAGs related to a gene, protein, miRNA, drug or disease. The ‘Basic Search’ results will return a list of PAGs related to the query. For the ‘Advanced Search’ option, users can query a list of genes to retrieve the most similar PAGs in the database.

**Figure 3.**
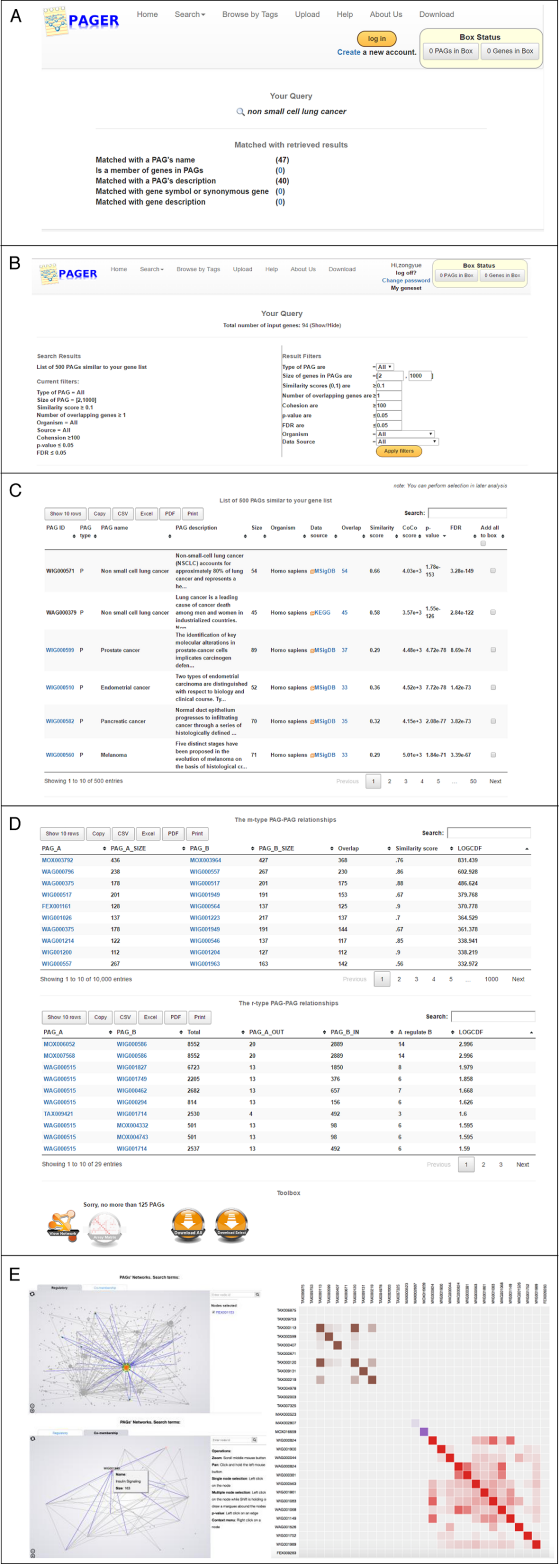
PAGER 2.0 Web Interface. (**A**) The refined result page searching by keyword. (**B**) The overall of retrieved PAG results by using a list of genes relevant to Non-Small Cell Lung Cancer. Statistical parameters and *nCoCo* score for filtering the results. (**C**) Results of the PAGs related to the query of genes relevant to Non-Small Cell Lung Cancer. (**D**) The m-type and r-type PAG-to-PAG relationships, (**E**) Visualization of the m-type and r-type PAGs networks and PAG-to-PAG similarity matrix.

**Figure 4.**
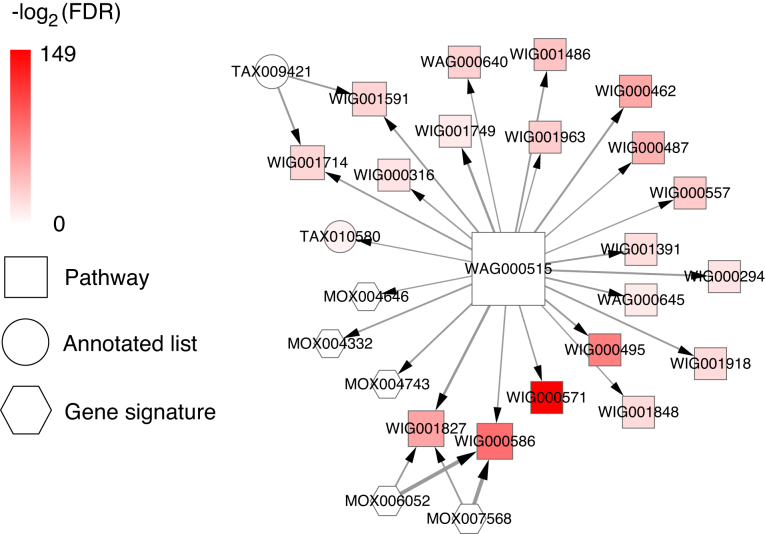
The r-type PAG-to-PAG's network of NSCLC study. The nodes represent the PAGs. The width of the edges denotes the score of r-type PAG-to-PAG's relationship. The node color represents the -log2(FDR) value of the PAGs in the NSCLC enrichment analysis. The size and shape of the nodes represent the degree and the type of PAGs, respectively.

To illustrate a use case example, we assume that a user is interested in finding PAGs that are related to ‘Non-Small Cell Lung Cancer’. The user can enter the keyword ‘Non-Small Cell Lung Cancer’ in the search box of PAGER 2.0, and the refined result page shows the relevant result by direct matching with the PAG's name, matching with the PAG's description in Figure 3A. In this example, there are 47 PAGs retrieved by matching with the names and 40 PAGs retrieved by matching with the descriptions. The user can click the PAGs to see the PAG detail's page. This feature allows user to quickly retrieve the relevant PAGs from different omic-levels (e.g. GWAS catalog PAGs show genetic variations, KEGG PAGs provide pathways and MSigDB PAGs present gene expression signatures) about the disease. Another example is a user can query a list of genes in PAGER 2.0 to identify related PAGs with the query. In Figure 3B, a list of 94 genes related to ‘Non-Small Cell Lung Cancer’ was used as the query for the ‘Advanced Search’ option. We set up the parameter ‘the type of PAG’ as ‘all’, ‘the size of PAGs’ as ‘[2–1000]’, ‘the similarity score’ ≥ 0.1, ‘the number of overlapping genes’ as ‘>1’, ‘the cohesion score’ ≥100, ‘FDR’ ≤ 0.05. Figure 3C shows the results of this query, where 500 PAGs (332 P-type + 25 A-type + 143 G-type) were returned. The results page also contains all the m-type and r-type PAG-to-PAG relationships (Figure 3D). The user can further filter the list of the results by refining the parameters (e.g. FDR, *P*-value, *nCoCo* scores, PAG size) tailored to their biological question and context. Finally, we also provide visualization options for the users to explore the PAG-to-PAG relationships in networks or matrix formats (Figure 3E). This visualization feature can assist user to navigate the PAG networks, and potentially uncover new insights and generate novel hypothesis through investigate the co-memberships of PAGs in the upstream and downstream networks. For example, in the NSCLC query, we were able to find the PAG:WAG000515 ‘RAF phosphorylates MEK’ as the largest hub PAG in the r-type PAG-to-PAG's network (Figure 4). Moreover, investigation of MEK inhibitors as the treatment for KRAS-mutant and BRAF-mutant NSCLC are actively studied in multiple clinical studies (41–44). In the advance search option, we could also retrieve the drugs that are highly relevant to the disease by searching the PAG term from DSigDB. In the example of the 94 ‘Non-Small Cell Lung Cancer’ gene list, we were able to find the drug ‘Gefitinib’ with FDR = 2.43e-26. Gefitinib is the FDA approved drugs for EGFR mutant non-small cell lung cancer patients. See USER MANUAL in the Supplementary File for details on using the PAGER 2.0 web interface.

### Gene prioritization using *RP-score*

**Figure 5.**
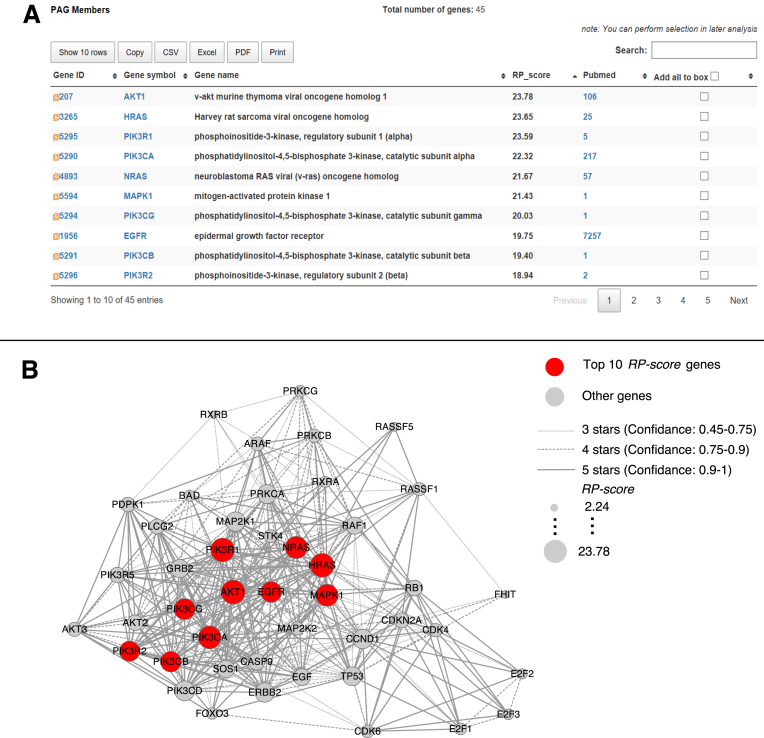
Gene prioritization with *RP-score*. (**A**) Top 10 genes in the ‘Non-Small Cell Lung Cancer’ PAG WAG000379 ranked by *RP-score*. (**B**) Genes with high *RP-score* (colored in red) are tightly connected in the protein–protein interactions. The size of the nodes represents the *RP-score*, and the width of the edges represents the confidence score for the protein–protein interactions as obtained from HAPPI-2 database source.

To illustrate the new intra-PAG gene prioritization feature in PAGER 2.0, we used the PAG named ‘Non-Small Cell Lung Cancer (NSCLC)’ (ID = WAG000379) as an example. Figure 5A shows a result of the top 10 genes (colored in red) with the size drawn in proportion to their RP-scores calculated in this PAG. Since the *RP-score* may incorporate either direct or indirect PPI information, genes such as EGFR and AKT1 that are positioned upstream of the NSCLC signaling cascades, which includes EGFR, RAS/MAPK and AKT/PI3K pathways, gained higher scores than other genes positioned downstream of the NSCLC signaling cascades. In Figure 5B, we show a network visualization plot, which we draw using the Cytoscape software version 2.83 with the data exported from PAGER 2.0 query results. The network visualization enables the user to gain insights on functionally significant genes within a PAG.

### Download of results and data availability

For every query performed, users can customize the results (columns) of interest in the PAG result page. The results can then be downloaded in common formats, including flat files (comma separated), Excel, and PDF. In addition, we also provide users the option to download the entirety of PAGs in PAGER 2.0 as a GSEA gene set (.gmt) file. The .gmt file format can be directly imported into GSEA to execute the program. Similarly, we allow bulk download of all PAG’s information, PAG-to-PAG relationship and gene–gene relationship contains in PAGER 2.0.

### New features of PAGER 2.0

The summary of the new features of PAGER 2.0 is in Table 3. We have implemented four content updates and four functional updates in PAGER 2.0.

**Table 3.** New features in PAGER 2.0

New features in PAGER 2.0	
**Contents**	• Gene prioritization in PAGs
	• Evidence of gene member in PAGs supporting from PUBMED
	• m-type and r-type PAG-to-PAG relationship detail, gene–gene interaction and gene–gene regulation in PAGs
	• New PAGER 2.0 GMT file for GSEA
**Functions**	• Bulk download of PAG’s information, PAG-to-PAG relationship, and gene–gene relationship
	• Marks the suspected PAGs with comments and submit to our system for curation
	• Uploading system updates: supports file uploading
	• Search button to filter the content in the results

## CONCLUSION

Genes act in concert to drive various biological processes in a complex biological system. High-throughput omics technologies are generating measurements for these biological systems at an unprecedented pace. GNPA provide a powerful approach to analyze and interpret these ‘omics’ datasets to reveal the underlying molecular mechanisms of gene–gene interactions. To facilitate and support GNPA methods, we have developed PAGER 2.0, a comprehensive database that integrates heterogeneous gene-sets, molecular signatures, and pathway/network modules into a unified framework. In PAGER 2.0, we extended the concepts of PAGs and imported new PAGs from 10 sources that increased the amount of PAGs by almost three times. The significant improvement in heterogeneous PAGs definition can assist researchers in acquiring comprehensive insight (diseases, gene expression signatures, drug, miRNA, gene, protein, pathways, functional annotation, tissue-specific expression) of GNPA. The m-type and r-type PAG-to-PAG relationships have been increased by four times. The increased coverage of PAG-to-PAG relationships provides the comprehensive linking between the omics data. The new PAGs' quality measurement, the *nCoCo* score is designed for assessing the biological relevance, and gene ranking score (*RP-score*) is developed to rank the gene member in PAGs, which raises researcher’s interests on network analysis level. The *nCoCo* score and gene prioritization enable the user to filter the genes in GPNA. In summary, we have updated PAGER 2.0 with new features and data (PAGs coverage and size) that could help users to gain more significant and quantitative biological insights in analyzing their omics datasets. We believe PAGER 2.0 will be a powerful tool and data resource that facilitates the use of GPNA in various omics data and network biology studies.

## Supplementary Material

Supplementary Data
