# Supplementary material for: PAGER 2.0: an update to the pathway, annotated-list and gene-signature electronic repository for Human Network Biology
Source: Nucleic Acids Res. 2017 Nov 8;46(Database issue):D668–76. doi: 10.1093/nar/gkx1040 (PMC5753198; doi:10.1093/nar/gkx1040)
Supplement: Supplementary Data [file gkx1040_supp.zip › nar-02489-data-e-2017-File007.docx]

## Supplemental Files

**PAGER 2.0: An update to the Pathways, Annotated-list, and Gene-signature Electronic Repository for Human Network Biology**

Zongliang Yue^1^, Qi Zheng^3,1^, Michael T. Neylon^4^, Minjae Yoo^2^, Jimin Shin^2^, Zhiying Zhao^5^, Aik Choon Tan^2^ and Jake Y. Chen^1^*

**Table of Contents**

**Supplementary Materials and Methods…………………………… 2**

**Supplementary Results …………………………… 5**

**PAGER 2.0 USER MANUAL …………………………… 6**

**Supplementary Figures …………………………… 10**

**Supplementary Tables …………………………… 11**

### Supplementary Materials and Methods

**Data Sources**

The 24 data sources including ten new data sources for PAGER 2.0 is shown in **Table S1**. Data was collected from a Phenome Wide association study (1) conducted at Vanderbilt University. This study prepared a PheWAS catalog, which contains the PheWAS results for 1,358 single-nucleotide polymorphisms (SNPs present in the NHGRI GWAS Catalog in 13,835 European-ancestry individuals from five sites of the Electronic Medical Records and Genomics (eMERGE) network. A total of 1,358 PAGs were created from the PheWAS data thus obtained. Gene sets were obtained from an Isozyme study (2) and mapped to PAGs by grouping genes to unique Isozyme IDs. There are 491 PAGs from Isozyme. Drugs and small molecule related changes in gene expression were also mapped to PAGs by including gene sets from DSigDB. Each DSigDB gene set consists of a list of target genes of a compound. Entrez Gene IDs is used as IDs to map across different databases. DSigDB (3) currently holds 22,527 gene sets, and consists of 17,389 unique compounds covering 19,531 genes. DSigDB gene sets are acquired through both automatic computational methods and manual curation. Immunological data from a recent study called ImmuneSigDB (4) has been included in PAGER. ImmuneSigDB is a manually annotated compendium of 4,872 gene-sets from diverse cell states, experimental manipulations, and genetic perturbations in immunology. miRNA target interaction information from high-throughput sequencing experiments were collected from MicroCosm (5) - an online database for miRNA target prediction and functional annotations, mirTARbase (6) - more than three hundred and sixty thousand miRNA-target interactions (MTIs) collected by manually surveying pertinent literature after NLP of the text systematically to filter research articles related to functional studies of miRNAs, and TargetScan (7) - predicted biological targets of miRNAs by searching for the presence of conserved 8mer, 7mer, and 6mer sites that match the seed region of each miRNA. All the targets in the three miRNA target sources collected and 9,407 PAGs were formed by grouping genes having the same miRNA target from. The GO term has been collected from amiGO (8) database. The genes from Homo Sapiens that share the same functional term in biological processes, cellular components, and molecular functions were grouped in the same gene set. There were 11,750 PAGs from amiGO. The protein families are performed by multiple sequence alignments and hidden Markov models (HMMs). There were 1,167 PAGs collected from Pfam (9). To determine tissue-specific gene expression in each PAG, we obtained RNA-seq data of various normal tissues from Genotype-Tissue Expression (GTEx) project (<http://www.gtexportal.org/>) (10). We extracted the genes within the top edges (with a posterior probability > 0.95) from the Genome-scale Integrated Analysis of gene Networks in Tissues (GIANT, <http://giant.princeton.edu/>). Using the genes extracted from GIANT for each tissue-specific, we filtered the gene expressions with RPKM > 10 and considered these as tissue-specific genes. In total, we extracted 53 tissue-specific gene expressions, with the mean 56 genes per tissue in FPKM ≥ 50 (in the range of 2 – 819), and with the mean 40 genes per tissue in FPKM ≥ 100 (in the range of 2 – 452). These tissue-specific genes were highlighted in the browser.

***nCoCo* score calculation**

The PAGs quality metric—*CoCo score* (a Cohesion Coefficient derived from the measure of statistically significant coverage of gene-gene functional correlations in gene pairs or gene trios) in PAGER 1.0 didn’t consider the bias of PAG size. We improve the *CoCo* score to a new PAG size-normalized new quality metric. The new score—normalized *CoCo (nCoCo)* rescales *CoCo* scores based on polynomial regression models to eliminate the PAG size bias in *CoCo* scores used in PAGER 1.0. In the PAGER 1.0, we applied the HAPPI-2 database to recalculate the Cohesion of protein-protein Interaction (*CoI*) and the Cohesion of Triangles (*CoT*) score based on the hypergeometric cumulative distribution function (CDF) value using the formula:

$$CoI(p)=sign\left( \frac{n}{k}-\frac{N}{K} \right)\times-{log}_{10} \left( \sum_{t=k}^{min(n,K)} \frac{\left( \begin{aligned} K \\ t \end{aligned} \right)\left( \begin{aligned} N-K \\ n-t \end{aligned} \right)}{\left( \begin{aligned} N \\ n \end{aligned} \right)} \right)$$

in which *N=(|Π|×(|Π|−1))/2*. *N* is the number of theoretical PPIs in *Π*, *K = |IΠ|* is the number of actual PPIs in the referenced database, *n=(|PAG_i|×(|PAG_i|−1))/2*. *n* is the theoretical number of PPIs inside PAG i, and *k = |Ii|* is the number of actual PPIs inside PAG i. The sign function compares the expected PPI count ratio *K/N* and PAG i’s PPI count ratio *k/n*. It returns 1 for over-representation if the PPI count ratio inside PAG i is more than the expected ratio, and returns −1 for under-representation if the interaction ratio inside PAG i is less than the expected ratio. For PAGs containing no PPIs, we will not calculate a *CoI*. A high positive *CoI* score implies that genes inside the PAG are strongly linked while a high negative *CoI* score implies that genes inside the PAG are impossibly linked. Randomly generated PAGs should have a mean *CoI* close to 0. We developed *CoT* to measure the statistical significance of observing a given number of triangles (PPIs forming a connected loop with three exact nodes/edges) among all genes within a PAG. Similar to *CoI*, *CoT* is also calculated using the same hypergeometric distribution cumulative distribution function (hence we will not repeat here) but with different parameters for counting PPI triangles instead: *N=(|Π|×(|Π|−1)×(|Π|−2))/6*. *N* is the number of theoretical triangles in *Π*, *K = |TΠ|* is the number of actual triangles in the referenced database, *n=(|PAG_i|×(|PAG_i|−1)×(|PAG_i|−2))/6*. *n* is the theoretical number of triangles inside *PAG_i*, and *k = |Ti|* is the number of actual triangles inside *PAG_i*. When compared with *CoI*, it has similar characteristics for highly positive *CoT* or highly negative *CoT* cases. For PAGs containing no PPIs, we will not calculate a *CoT*. A high positive *CoT* score implies that genes inside the PAG are strongly linked while a high negative *CoT* score implies that genes inside the PAG are impossibly linked. Randomly generated PAGs should have a mean *CoT* close to 0.

To compute the *nCoCo* scores, first, we applied the HAPPI-2 database to recalculate the *CoI* and *CoT* scores using the hypergeometric cumulative distribution function (CDF). Second, we build the multi-box plots using the bin size of 2^n^ of the PAG size and used the median to represent the value in each bin and applied the polynomial function to find the regression of the *CoI* score vs PAG size.

*CoI(p)=Sz(p)^2^*a+ Sz(p)*b*,

where *Sz(p)* is the size of the PAG p, and the *CoI(p)* is the *CoI* score of the PAG p.

Third, we calculated *nCoCo* based on the formula:

*nCoI(p)=med(PAGn)*CoI(p)/ [Sz(p) *a+ Sz(p)^2^*b]，*

where *med(PAGn)* is the median of all PAGs.

Fourth, the *nCoCo* score is calculated by the sum of the normalized interactive score *nCoI* and normalized triangle score *nCoT*:

*nCoCo(p)=nCoI(p)+nCoT(p)*

**Gene prioritization using PAG.**

The input file of *RP-score* rank is the gene list. The output file is the gene list ordered by the PAGER rank score. We use *RP-score* to calculate and assign the gene weight in every PAG.

$$RP-score=e^{k \times ln(\sum_{p,q\in PAG} conf\left( p,q \right))-ln(\sum_{p,q\in PAG} N\left( p,q \right))}$$

where *p* and *q* are the indexes of proteins from the selected module, *k* is a constant (*k*=2 in this study). The term *conf(p, q)* is the interaction confidence score assigned by HAPPI-2, where *conf(p, q)* is between 0 and 1. *N(p, q)* holds the value of 1 if protein *p* interacts with *q* or the value of 0 if protein *p* does not interact with *q*. Then, we rank the genes by the *RP-score* and seek the potential important genes in the top rank list.

### Supplementary Results

**PAG identifiers**

The identifier of the PAGs is based on the component of three letters and 6 digits. The first position has 9 different letters standing for the PAG types. In **Table S2**, the type of the PAG, the source of the PAG and T is for the ontology data, W is for the pathway data, G is for the genomic or epigenetic data, F is for the functional data, P is for proteomics data, M is for chemical perturbation data, B is for metabolomics data, R is for PAGER data, and N is the genomic single gene. The second position had 5 different letters standing for the PAG derivation method. E is for experimentally derived data, O is for computationally predicted data, A is for curated from literature data, U is for unknown or uncharacterized data, and I is for known missing curation data. The position three had 9 different letters for the PAG relationship details. X is for the PAGs with no relationship mapped, I is for the PAGs with some interactions, J is for the PAGs with interaction and parameters of interaction, G is for the PAGs with some regulatory data, H is for the PAGs with some regulatory data and parameter of regulation, R is for the PAGs with chemical reaction, S is for the PAGs with some chemical reaction and parameter of reactions, M is for the PAGs with model data (mixture of regulatory and reaction data), and P is for the PAGs with parameterized model data (mixture regulatory and reaction data).

**PAG size distribution**

In **Fig S1**, the PAG size distribution is grouped by the type of the PAG and derivation method. All the experimental result (*.E.*) are following power law except for the functional genomics data (*F..*) which is a normal distribution. Since the functional genomics data is collected from the PAGs relevance to gene signature to reveal functions in a specific disease, the size of this type of data always has some size preference (*FO.* median: 62, *FE.* median: 200, *FA.* median: 200). The *WI.* was known as the pathway data and missing curation data have the size preference (*WI.* median:32) since the pathway is pre-acknowledge gene-set with certain biological function. The *GO.* and *GA.* are the genomics or epigenetic data have size preference (*GO.* median:114, *GA.* median:72) since the epigenetic sources like Phewas having a certain amount of biomarkers for detecting the diseases.

***nCoCo* score**

*nCoCo* score is calculated by summarizing the rescaled *CoI* and rescaled *CoT*. The size effect of *CoI* and *CoT* is shown in **Fig S2** (a, c). The PAG size and *CoI* score has a polynomial relationship as the linear regression function *log_2_[CoI(p)]* = *0.66*log_2_[Sz(p)] - 0.37* possessing the good fitness (R square = 0.80). The PAG size and *CoT* score has a polynomial relationship as the polynomial regression function *log_2_[CoT(p)] = 0.05*log_2_[Sz(p)]^2^+0.36*log_2_[Sz(p)]* possessing the very good fitness (R square = 0.95). After the rescaling of the *CoCo* score, the *nCoI* and *nCoT* relationship to the PAG size relationship is shown in **Fig S2** (b, d). The distribution of the *CoI*, *CoT* and *CoCo* score is shown in **Fig S2** (e), and the distribution of the *nCoI*, *nCoT* and *nCoCo* is shown in **Fig S2** (f). Comparing the *CoCo* and *nCoCo*, the distribution shifting to the left side in the *nCoCo* score indicates most of the size inflated *CoCo* score has been eliminated by using polynomial regression normalization.

### PAGER 2.0 USER MANUAL

The user guideline of PAGER 2.0 is shown in **Fig S6**.

**Home Page**

The PAGER 2.0 home page can be accessed at http://discovery.informatics.uab.edu/PAGER/. This page is a PHP interface for the PAGER 2.0 database, which is an Oracle 12g database. The home page includes a basic search as well as links to ‘Advanced Search’, ‘Browse by Tags’, ‘Upload’, ‘Help’, ’About Us’ and ‘Download’. It also includes the ‘PAG Box’ and account login.

**Basic Search and Advanced Search**

The basic search on the home page allows a user to enter terms, such as a disease name, a gene symbol or PAG name, as well as drug name and miRNA. The advanced search allows a user to enter a list of gene symbols with options to filter the results. These options are also available on the results page, which is the same for both searches. It gives the options to filter by PAG size, similarity score, number of overlapping genes, normalized Correlation Coefficient score(CoCo score), p-value, FDR, organism, and data source. If a user enters a list of genes, PAGER 2.0 returns related PAGs, which contain genes in the list, CoCo score (using CDF to substitute pmf of the previous version), p-value and False Discovery Rate (FDR). If users enter terms, PAGER 2.0 returns genes and PAGs, which match with the terms. In addition, users can view details about a particular PAG by clicking on it.

**Gene and PAG Box**

A status of ‘PAG Box’ and ‘Gene Box’ is displayed on the top right of the page. Users can add PAGs to the ‘PAG Box’ in order to construct an r-type PAG-to-PAG and an m-type PAG-to-PAG relationship and a PAG network that can be viewed. Users add genes to ‘Gene Box’ in order to construct gene regulation network and gene interaction network.

**Browse By Tags**

Browse by tag allows a user to browse PAGs based on the disease with which they are associated. These general groups of diseases can be clicked on to return a list of associated PAGs. The general groups of diseases can be expanded a couple more levels to show a list of more specific diseases within that group. Each of these levels can be clicked on to view a list of associated PAGs as well.

**Upload**

Upload allows a user to select a species and enter his or her own gene symbols submitting to PAGER 2.0. At the same time, a user can upload a file involving gene symbols or gene IDs, which will be evaluated by PAGER 2.0. If the PAGs that uploaded by the user are helpful, the PAGs will be entitled by the user’s name and can be browsed by all other users.

**Help**

The ‘Help’ page offers explanations and examples.

**Download**

Download allows a user to download our latest version of PAGs.

**Account Login and Registration**

There is an account login and account registration at the top of the page. This allows a user to create a login for the site so they can create their own gene sets and save them.

**Tool Box**

**Network View**

The Network View can be accessed either on the bottom of the results page of the search or from within the ‘PAG Box’ or ‘Gene Box’. The network visualization uses cytoscape.js, an open source-graph library.

In order to construct PAG networks in the ‘PAG Box’, users go into it by clicking on the status on the top right of the page. On the ‘PAG Box’ page, users can construct both m-PAG and r-PAG networks of PAGs in the box. Compared to the last version, we added more m-PAGs and r-PAGs, which provides more similarity and relationship among PAGs and enlarge the PAG-to-PAG networks. Related PAGs of a particular PAG were separated into three groups: co-membership m-PAGs, upstream, and downstream r-PAGs. The user can also construct PAG networks of the related PAGs, an expanded PAG networks. On the detail page, users can view gene networks of a PAG.

Both PAG and gene networks were displayed as interactive networks. Users can click on a node in networks to see more details or links to the gene or PAG detail page. In addition, users can select a PAG in the expanded PAG network and add the PAGs to ‘PAG Box’. To sum up, PAGER can be used for constructing multiple levels of biological networks, gene, and PAG networks, to gain insights into underlying biological mechanism. For example, users enter a list of disease genes and PAGER returns a list of related PAGs. Users then add the disease-related PAGs to ‘PAG Box’ and construct disease-specific PAG networks. These PAG networks can be used to reveal insights into the disease. More use cases can be found on the ‘Help’ page.

**Matrix View**

At the bottom of the results page of a search, there is a ‘View Matrix’ button. This matrix is visualized with D3.js, a JavaScript library for manipulating documents based on data. This matrix representation of PAG similarity is an adjacency matrix in which each cell represents an edge from vertex i to vertex j. Vertices represent PAGs, which are the result of using the search. The color intensity of a cell represents the similarity score of the two PAGs, which is a measure of how many of the same genes the two sets share. The matrix has a default view of sort by name, which sorts alphabetically starting from the origin at the top left. There are two other sort options of frequency and cluster. Frequency will sort by the PAGs that share genes with most other PAGs to those with no shared genes. The cluster is another sorting option that will rearrange the matrix to cluster PAGs by the group, which is represented by the different colors. In PAGER 2.0, the PAGs increase by 119%, which are combined with previous PAGs in the last version and we use CDF to substitute pdf, which is more precise to show the similarity between PAG and PAG.

**Check box page**

In this page, a user can get the total number of PAGs searched as well as the current filter condition, which can be changed at any time. A user can also get the results of PAG ID, PAG name, PAG description, size, Pubmed reference, organism and data source. If a user is interested in any PAG, he or she can click ‘Add to the box’ to save the PAG name to the PAG box. If a user wants to get the details of the PAG, click the PAG ID or ‘more detail’ in PAG description.

**Gene/drug detail page**

In this page, a user can get the genes or drug targeted genes of the PAG searched. What is more, he or she can get the gene ID (link to NCBI), gene symbol, gene name, source and RP score (from HAPPI-2 dataset instead of HAPPI-1 of the last version). The number of the source means how many times this gene was mentioned in papers from NCBI. *RP-score* means the representative of the gene in the PAG. The larger the RP score, the more important it is in the PAG. Clicking gene name, a user can get the upstream regulators, downstream regulators and associated regulators of this gene.

In PAGER 2.0, we provide more evidence of genes from NCBI, which maximizes the reliability. At the same time, a user can get the interaction of genes in the PAG searched, which are ranked by scores. The Interaction data from HAPPI-2 are 3 Star or greater rating and we provide a link to HAPPI-2 dataset to do the further analysis.

A user can also get the regulation of genes, such as activation, inhibition, phosphorylation etc.

All the data can be copied or downloaded as .xls file or PDF file.

##### Supplementary Figures

**Figure S1. The PAG size distribution grouped by the type of the PAGs.** The column stands for the first letter of the PAG identifier and the row stands for the second letter of the PAG identifier. The cells are filled by the PAG size distribution according to the PAG type using the PAG information from **Table S2**.

**Figure S2. PAGER 1.0 and PAGER 2.0 *CoI* and *CoT* normalization.** (a) The relationship of PAG size and *CoI* score, (b) the relationship of PAG size and *nCoI* score, (c) the relationship of PAG size and *CoT* score, (d) the relationship of PAG size and *nCoT* score, (e) the *CoI*, *CoT*, *CoCo* score distribution of PAGER 2.0, (f) the distribution of *nCoI*, *nCoT*, *nCoCo* score in PAGER 2.0.

**Figure S3. The comparison of *nCoCo* score at the cumulative percentage of 50% (*CP50*) from 10 sources and the baseline of PAGER 2.0 *nCoCo*.** The *nCoCo* score at *CP50* measures the quality of the PAGs from the same source. The PAGER 2.0 *nCoCo* score at *CP50* is used as the baseline (red line).

**Figure S4. *nCoCo* score cumulative percentage grouped by type.** The column stands for the first letter of the PAG identifier and the row stands for the second letter of the PAG identifier. The cells are filled by the PAG size distribution according to the PAG type using the PAG information from **Table S2**.

**Figure S5. (a)The m-type PAG-to-PAG CDF score distribution and (b) the r-type PAG-to-PAG CDF score distribution**

**Figure S6. The pages of PAGER 2.0 for usage.**

#### Supplementary Tables

**Table S1: Statistics of the Data Sources in PAGER 2.0.**

| **Data Type** | **Data Source** | **Descriptions** | **Number of PAGs** |  |
| --- | --- | --- | --- | --- |
|  |  |  | PAGER 1.0 | PAGER 2.0 |
| Diseases | GAD | Genetic Association Database | 1,679 | 1,679 |
|  | GWAS Catalog | A Catalog of Published Genome-Wide Association Studies provided by NHGRI | 1,574 | 1,238 |
|  | Phewas | Phewas database collects the data from human disease results from complex interactions between genes and environmental risk factors, and that variants from a few (<20) susceptibility genes variants are responsible for >50% of this disease burden. |  | 1,358 |
| Gene Expression Signatures | MSigDB | The Molecular Signatures Database is a collection of annotated gene sets for use with GSEA software | 10,295 | 10,295 |
|  | GeneSigDB | manually curated database and resource for analysis of gene expression signatures | 3,515 | 3,506 |
|  | MSigDB-ImmuneSigDB | Manual annotated compendium of gene sets from diverse cell states, experimental manipulations, and genetic perturbations in immunology |  | 4,872 |
| Drug-Gene Interactions | PharmGKB | The Pharmacogenomics Knowledge Base Bioinformatics portal which integrates protein information, databases and research tools for researchers and students | 102 | 102 |
|  | DSigDB | DSigDB organized drugs and small molecules related gene sets into four collections based on quantitative inhibition and/or drug-induced gene expression changes data. |  | 22,527 |
| Genes | Genome Data | Gene from official uniprot ID exclude single gene PAG from PAGER | 15,161 | 18,492 |
| Proteins | Protein Lounge |  | 393 | 388 |
|  | Pfam | Pfam is a large collection of protein families, represented by multiple sequence alignments and hidden Markov models (HMMs). |  | 1,167 |
|  | Isozyme | Collect enzymes that differ in amino acid sequence yet catalyze the same reaction. |  | 491 |
| Pathways | SPIKE | SPIKE is a database of highly curated human signaling pathways | 28 | 28 |
|  | WikiPathway | WikiPathways is an open, public platform dedicated to the curation of biological pathways by and for the scientific community | 202 | 200 |
|  | HPD-Reactome | Curated and peer reviewed pathway database | 652 | 532 |
|  | HPD-BioCarta | Source for biological pathways, displayed in a graphical format, mapping known genomic and proteomic relationships | 253 | 252 |
|  | HPD-PID | Biomedical database of human cellular signaling pathways. | 132 | 132 |
|  | HPD-KEGG | KEGG (Kyoto Encyclopedia of Genes and Genomes) is a collection of databases dealing with genomes, biological pathways, diseases, drugs, and chemical substances | 200 | 199 |
| miRNA-Gene Interactions | Microcosm Targets | MicroCosm Targets (formerly miRBase Targets) is a web resource developed by the Enright Lab at the EMBL-EBI containing computationally predicted targets for microRNAs across many species. |  | 851 |
|  | TargetScan | TargetScan predicts biological targets of miRNAs by searching for the presence of conserved 8mer, 7mer, and 6mer sites that match the seed region of each miRNA. |  | 390 |
|  | mirTARbase | miRTarBase has accumulated more than three hundred and sixty thousand miRNA-target interactions (MTIs), which are collected by manually surveying pertinent literature after NLP of the text systematically to filter research articles related to functional studies of miRNAs. |  | 3,684 |
| Tissue Specific Expressions | NGS Catalog | NGS Catalog: A database of next generation sequencing studies in humans | 69 | 56 |
|  | GTEx | FPKM over or equal to 50 or 100 in tissue specific expression from NGS. |  | 93 |
| Functional Annotations | GOA | Gene Ontology: the framework for the model of biology. The GO defines concepts/classes used to describe gene function, and relationships between these concepts. |  | 11,750 |

**Table S2 The type of data, derivation method and relationship detail reflected by the first 3 letters of PAG identifier.**

| **Pos #1:Type of data** | | **Pos #2:Derivation method** | | **Pos #3:Relationship details** | |
| --- | --- | --- | --- | --- | --- |
| T.. | From Ontology Data | .E. | Experimentally derived | ..X | No relationship mapped |
| W.. | From pathway data | .O. | Computationally predicted | ..I | Contains Some interactions |
| G.. | From Genomics/Epigenomics data | .A. | Curated from literature | ..J | Contains interactions + parameters of interactions |
| F.. | From functional genomics data | .U. | Unknown/Uncharacterized | ..G | Contains some regulatory data (may also include interactions) |
| P.. | From proteomics data | .I. | Known missing curation | ..H | Contains some regulatory data + parameter of regulations |
| M.. | From chemical perturbations data |  |  | ..R | Contains some chemical reaction data |
| B.. | From metabolomics data |  |  | ..S | Contains some chemical reaction data + parameters of reactions |
| R.. | From PAGER data |  |  | ..M | Contains model data (mixture regulatory and reaction data) |
| N.. | From genomics single gene |  |  | ..P | Contains parameterized model data (mixture regulatory and reaction data + parameters) |

**REFERENCES**

1. Denny, J.C., Bastarache, L., Ritchie, M.D., Carroll, R.J., Zink, R., Mosley, J.D., Field, J.R., Pulley, J.M., Ramirez, A.H., Bowton, E. *et al.* (2013) Systematic comparison of phenome-wide association study of electronic medical record data and genome-wide association study data. *Nat Biotechnol*, **31**, 1102-1110.

2. Aksoy, B.A., Demir, E., Babur, O., Wang, W., Jing, X., Schultz, N. and Sander, C. (2014) Prediction of individualized therapeutic vulnerabilities in cancer from genomic profiles. *Bioinformatics*, **30**, 2051-2059.

3. Yoo, M., Shin, J., Kim, J., Ryall, K.A., Lee, K., Lee, S., Jeon, M., Kang, J. and Tan, A.C. (2015) DSigDB: drug signatures database for gene set analysis. *Bioinformatics*, **31**, 3069-3071.

4. Godec, J., Tan, Y., Liberzon, A., Tamayo, P., Bhattacharya, S., Butte, A.J., Mesirov, J.P. and Haining, W.N. (2016) Compendium of Immune Signatures Identifies Conserved and Species-Specific Biology in Response to Inflammation. *Immunity*, **44**, 194-206.

5. Griffiths-Jones, S., Saini, H.K., van Dongen, S. and Enright, A.J. (2008) miRBase: tools for microRNA genomics. *Nucleic acids research*, **36**, D154-158.

6. Chou, C.H., Chang, N.W., Shrestha, S., Hsu, S.D., Lin, Y.L., Lee, W.H., Yang, C.D., Hong, H.C., Wei, T.Y., Tu, S.J. *et al.* (2016) miRTarBase 2016: updates to the experimentally validated miRNA-target interactions database. *Nucleic acids research*, **44**, D239-247.

7. Agarwal, V., Bell, G.W., Nam, J.W. and Bartel, D.P. (2015) Predicting effective microRNA target sites in mammalian mRNAs. *Elife*, **4**.

8. Gene Ontology, C. (2015) Gene Ontology Consortium: going forward. *Nucleic acids research*, **43**, D1049-1056.

9. Finn, R.D., Coggill, P., Eberhardt, R.Y., Eddy, S.R., Mistry, J., Mitchell, A.L., Potter, S.C., Punta, M., Qureshi, M., Sangrador-Vegas, A. *et al.* (2016) The Pfam protein families database: towards a more sustainable future. *Nucleic acids research*, **44**, D279-285.

10. Carithers, L.J. and Moore, H.M. (2015) The Genotype-Tissue Expression (GTEx) Project. *Biopreserv Biobank*, **13**, 307-308.
