# Supplementary material for: PAGER 2.0: an update to the pathway, annotated-list and gene-signature electronic repository for Human Network Biology
Source: Nucleic Acids Res. 2017 Nov 8;46(Database issue):D668–76. doi: 10.1093/nar/gkx1040 (PMC5753198; doi:10.1093/nar/gkx1040)
Supplement: Supplementary Data [file gkx1040_supp.zip › nar-02489-data-e-2017-File008.pdf]

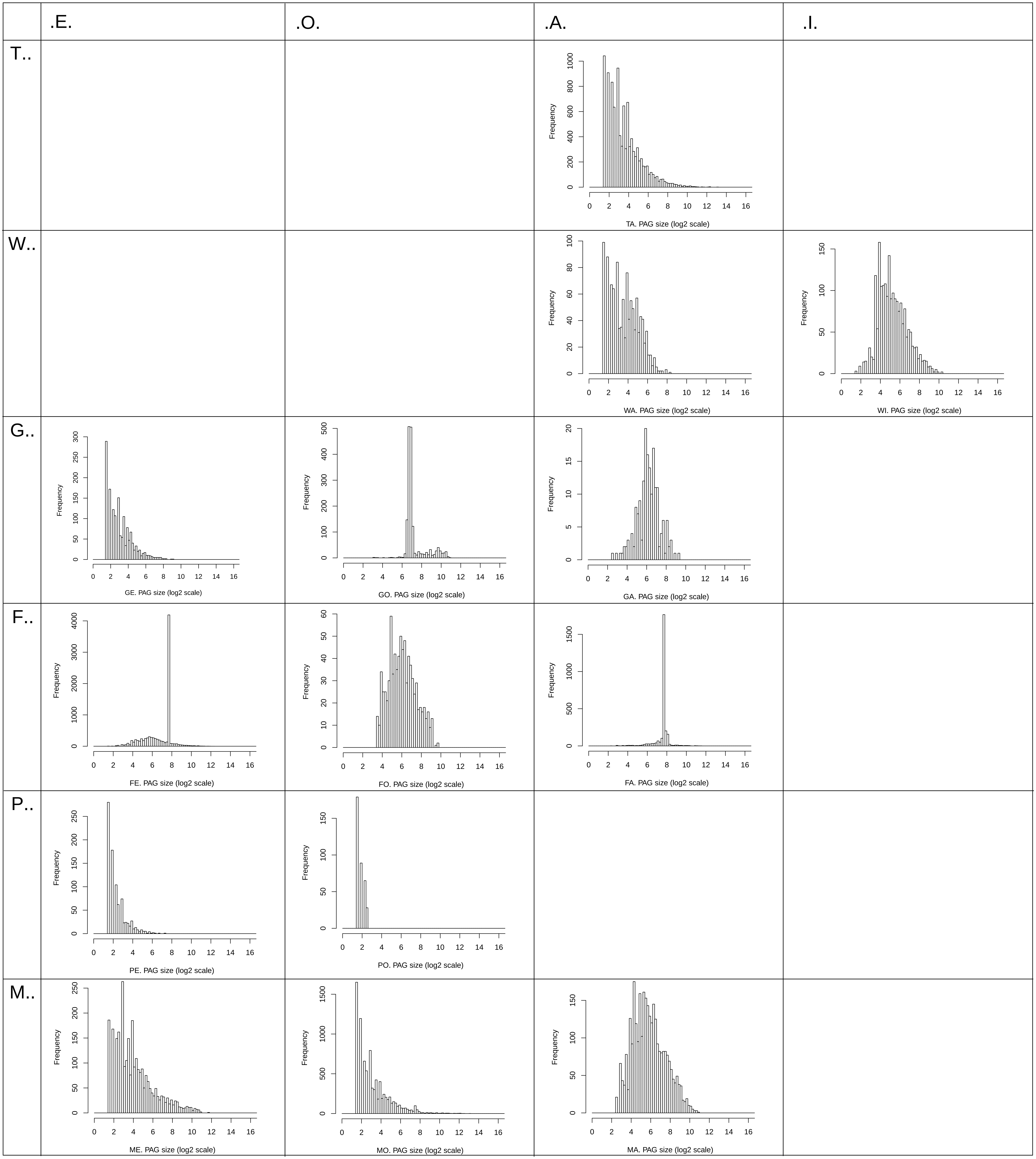
.E.
.O.
.A.
.I.
T..
W..
G..
F..
P..
M..
Frequency
TA. PAG size (log2 scale)
WA. PAG size (log2 scale)
WI. PAG size (log2 scale)
GE. PAG size (log2 scale)
GO. PAG size (log2 scale)
GA. PAG size (log2 scale)
FE. PAG size (log2 scale)
FO. PAG size (log2 scale)
FA. PAG size (log2 scale)
PE. PAG size (log2 scale)
PO. PAG size (log2 scale)
ME. PAG size (log2 scale)
MO. PAG size (log2 scale)
MA. PAG size (log2 scale)
