# Supplementary material for: PAGER 2.0: an update to the pathway, annotated-list and gene-signature electronic repository for Human Network Biology
Source: Nucleic Acids Res. 2017 Nov 8;46(Database issue):D668–76. doi: 10.1093/nar/gkx1040 (PMC5753198; doi:10.1093/nar/gkx1040)
Supplement: Supplementary Data [file gkx1040_supp.zip › nar-02489-data-e-2017-File013.pdf]

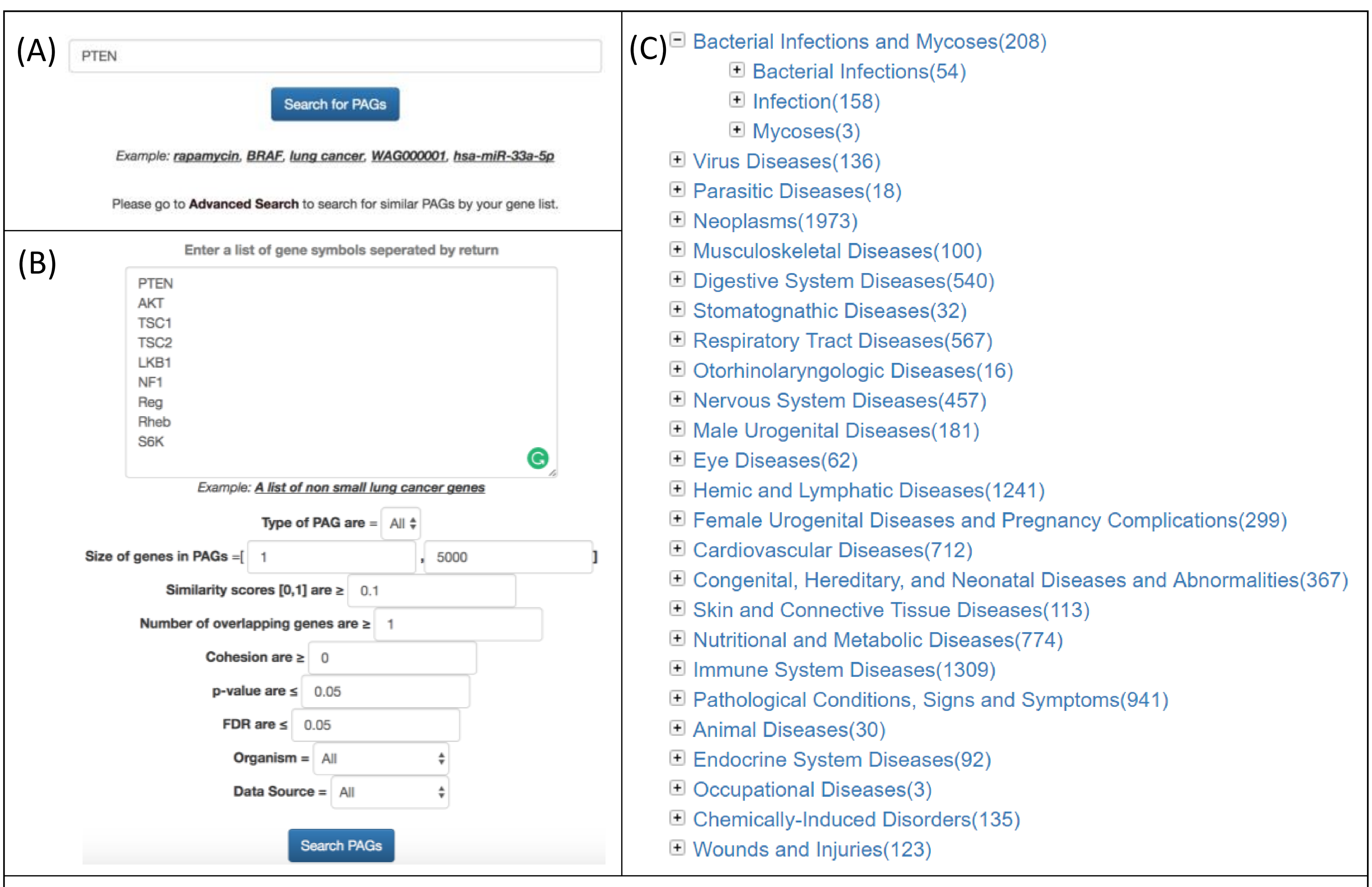

1. The input: keywords or gene symbol (A), a list of genes (B), or disease term (C)

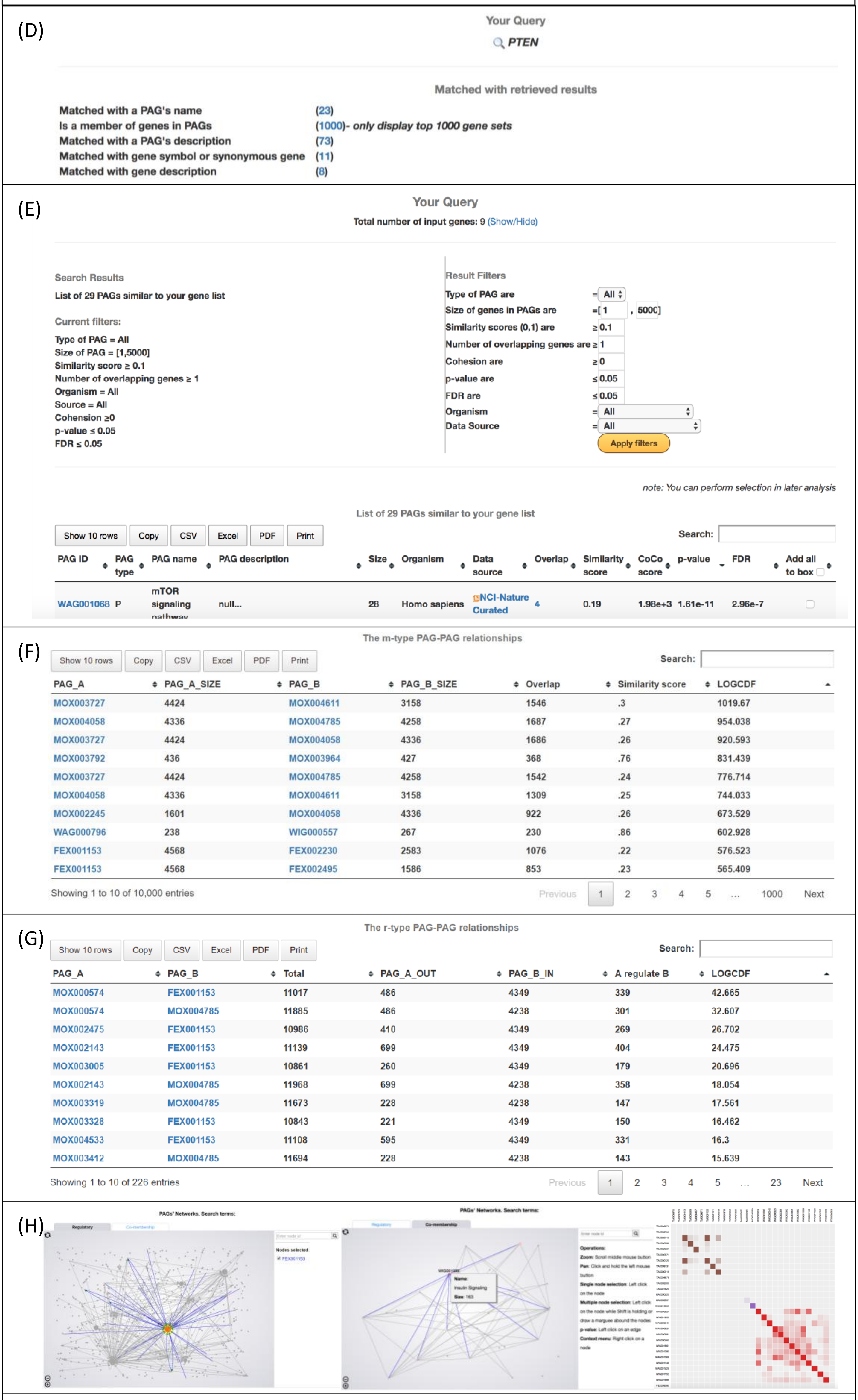

2. PAG-PAG relationships: refined result (D), related PAGs (E), the m-type PAG-PAG relationships (F), the r-type PAG-PAG relationships (G), and the m-type PAGs' network, the r-type PAGs' network and PAG-PAG similarity matrix (H).

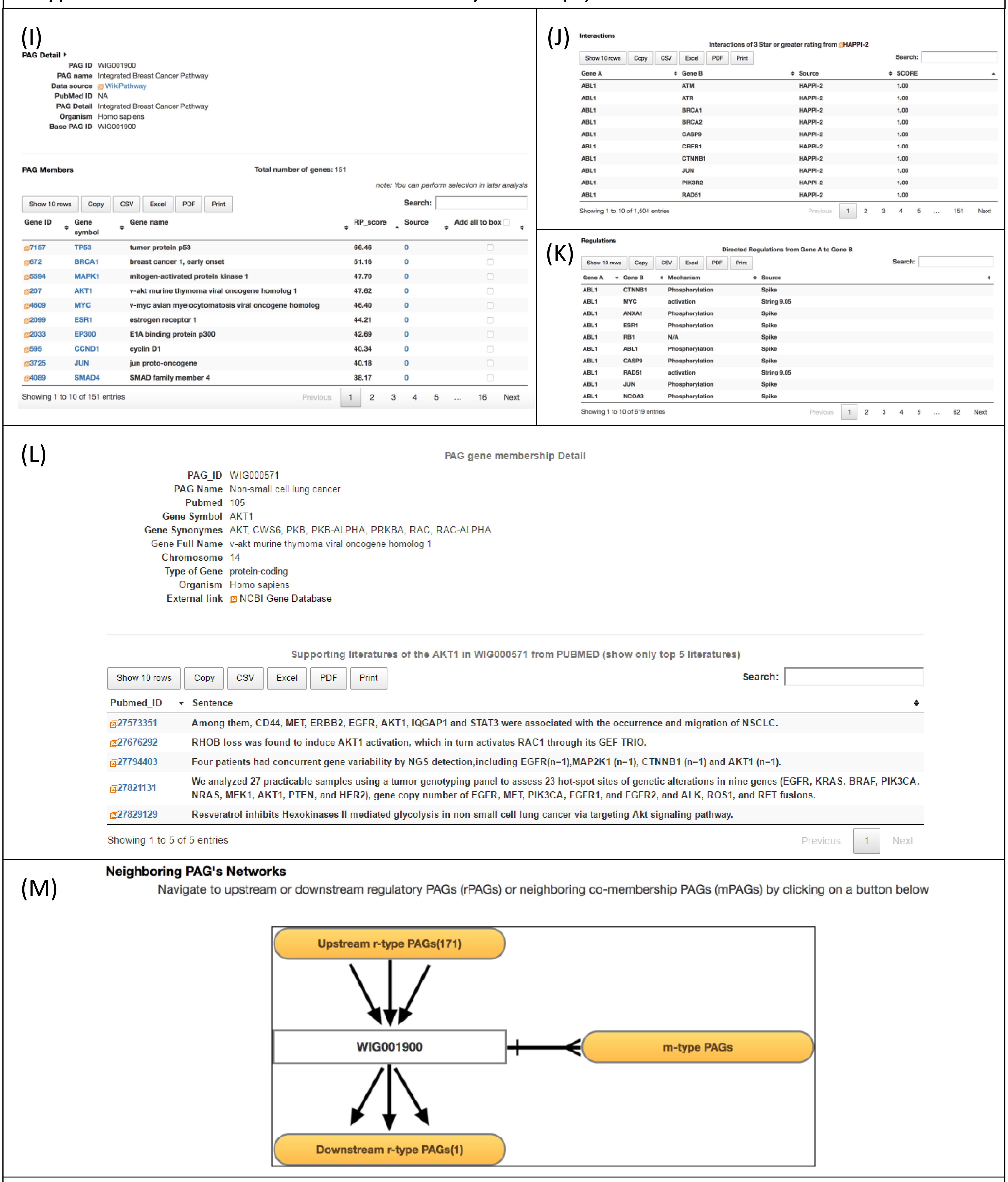

3. The PAG membership and detail: The PAG detail and gene membership (I), Gene-gene interactions in the PAG (J), Gene-gene regulations in the PAG (K), PAG membership detail and supporting evidence from PUBMED (L), and Up/down stream r-type PAGs and m-type PAGs (M).
