# Supplementary figures and images for: PAGER 2.0: an update to the pathway, annotated-list and gene-signature electronic repository for Human Network Biology
Source: Nucleic Acids Res. 2017 Nov 8;46(Database issue):D668–76. doi: 10.1093/nar/gkx1040 (PMC5753198; doi:10.1093/nar/gkx1040)

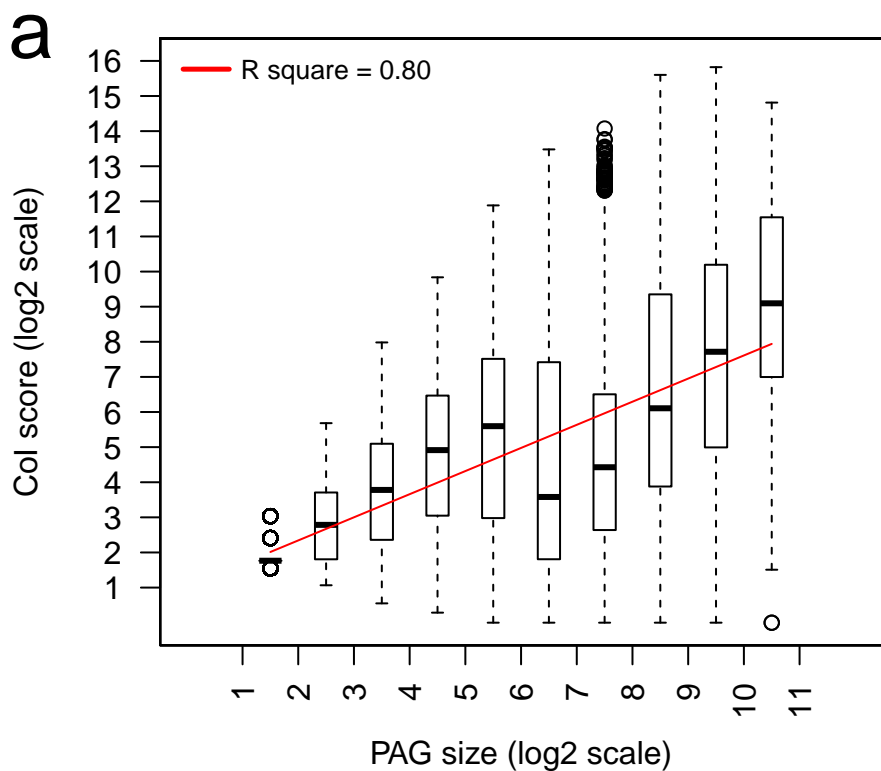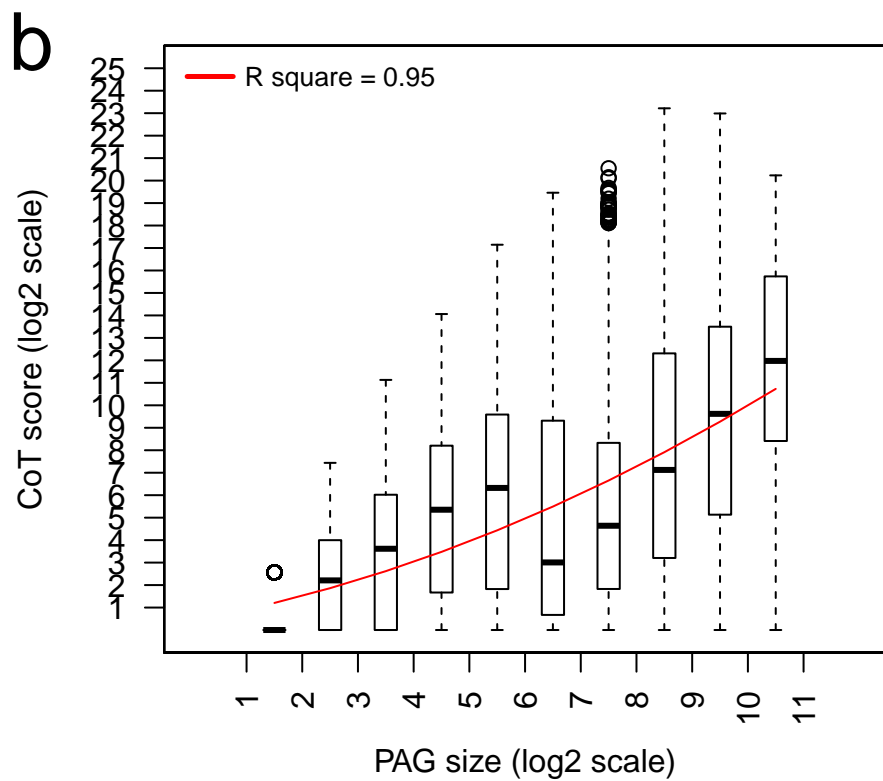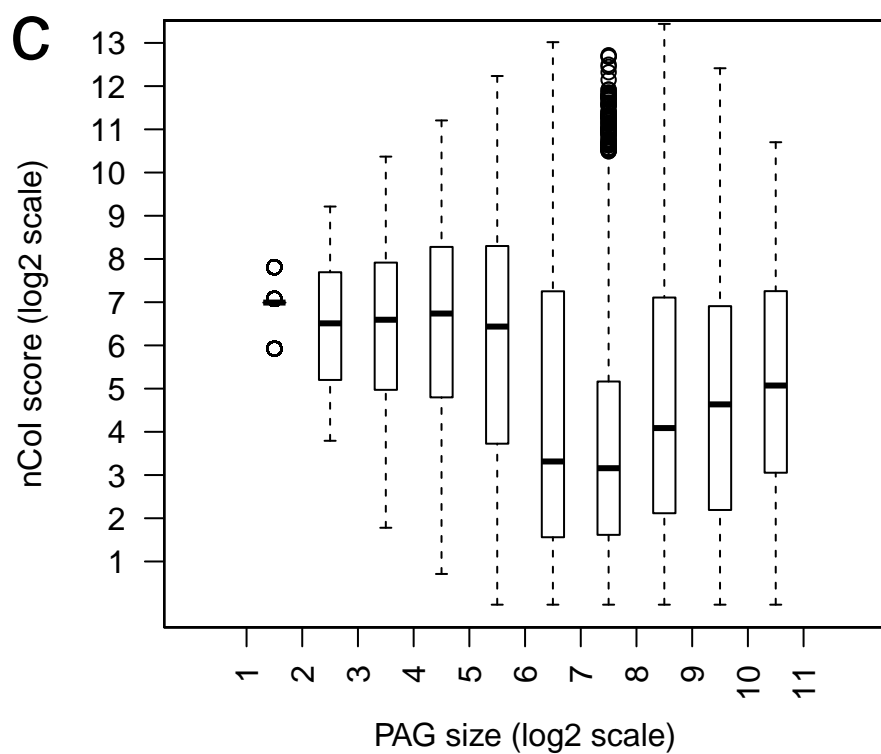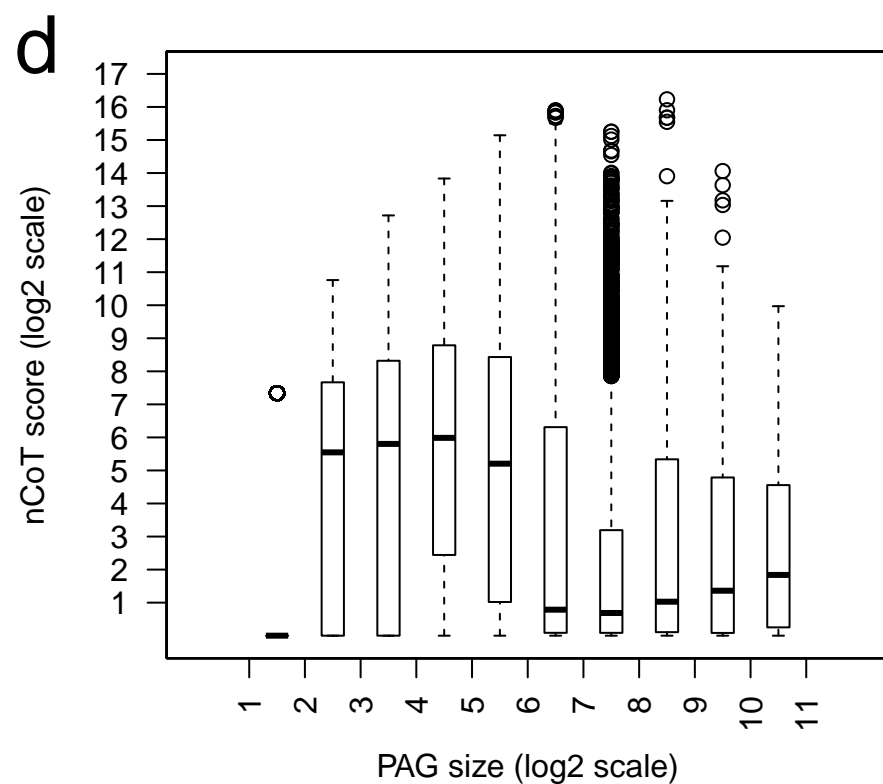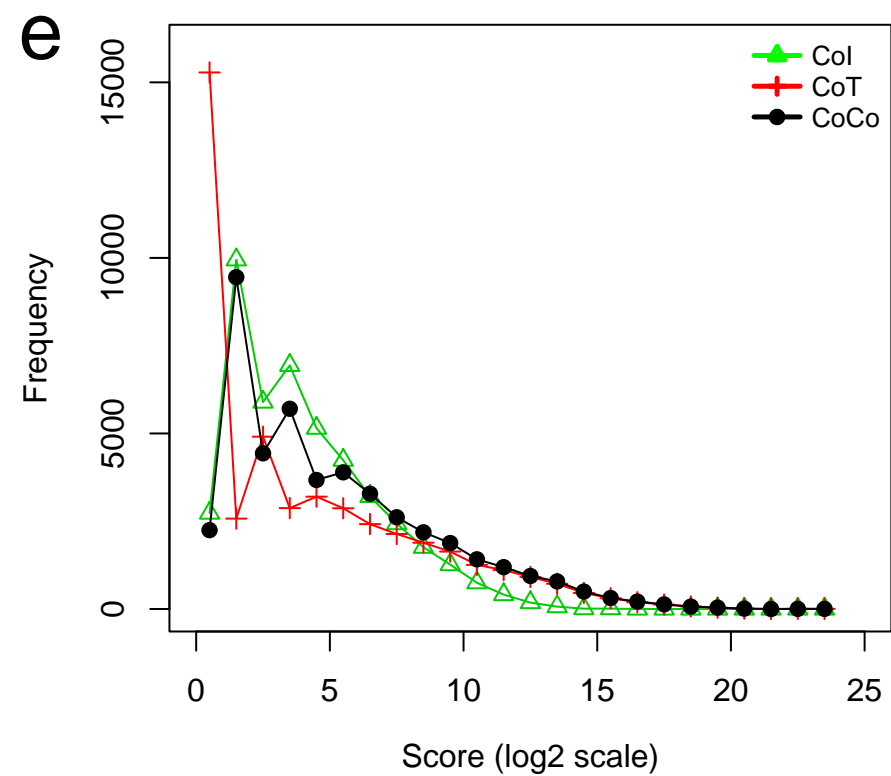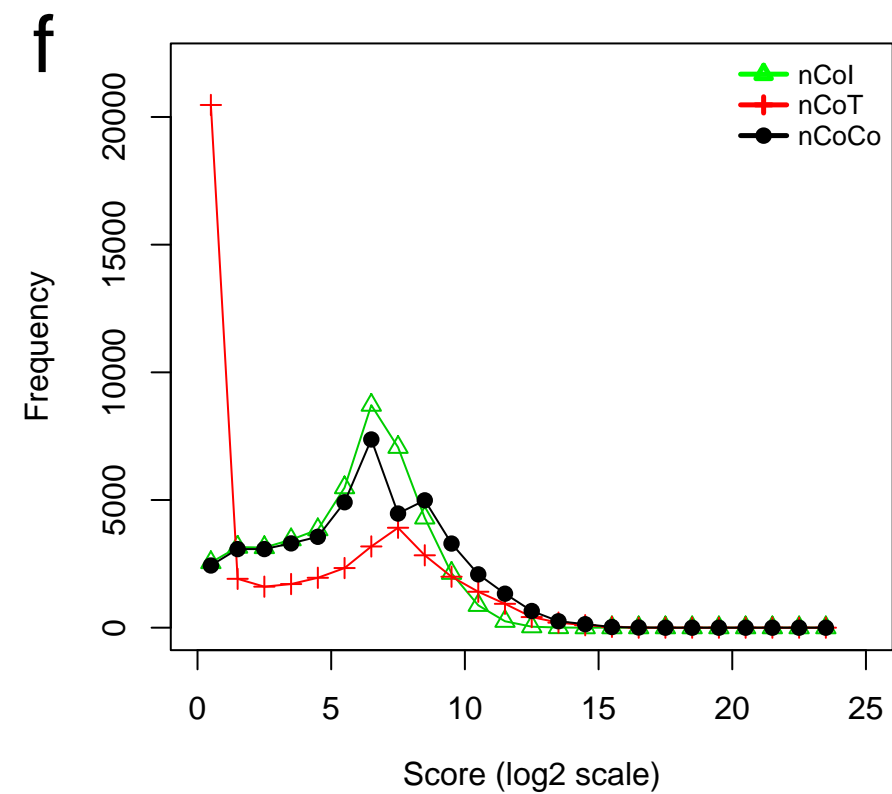

Supplement: Supplementary Data [file gkx1040_supp.zip › nar-02489-data-e-2017-File009.pdf]

nCoCo of cumulative percentage at 50% (CP50)

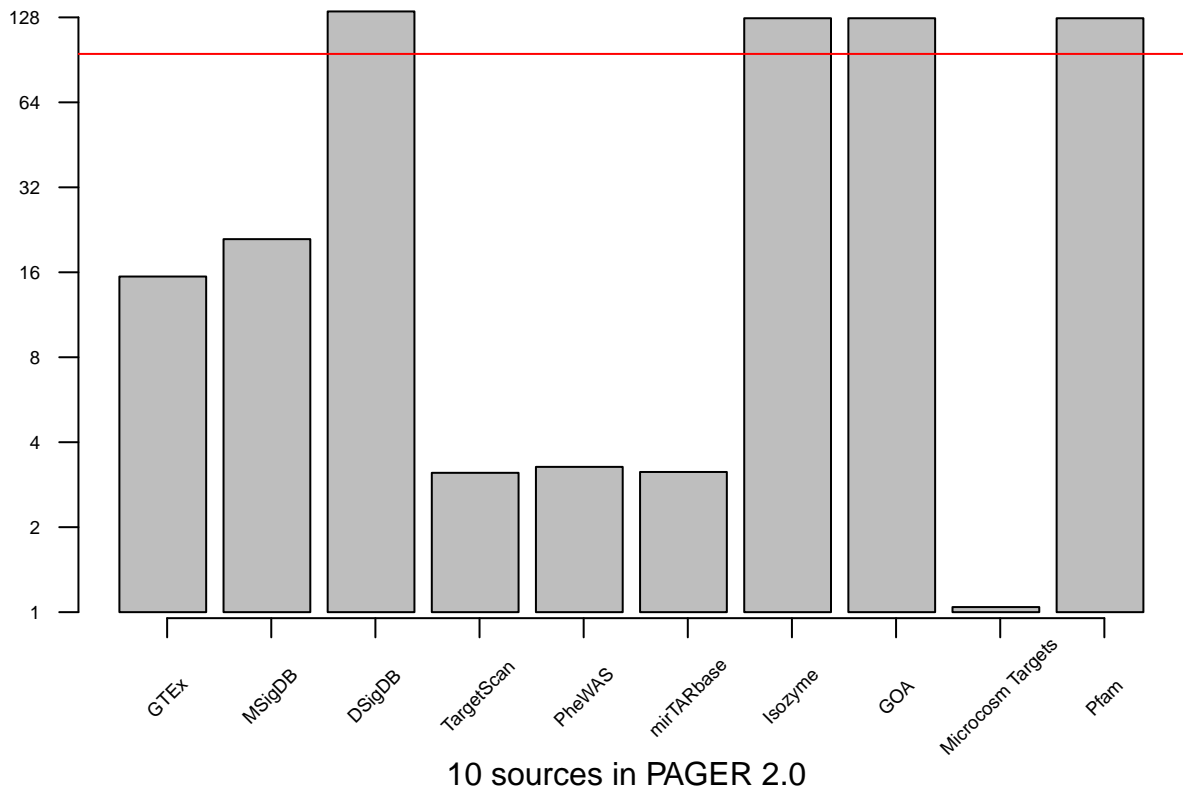

Supplement: Supplementary Data [file gkx1040_supp.zip › nar-02489-data-e-2017-File010.pdf]

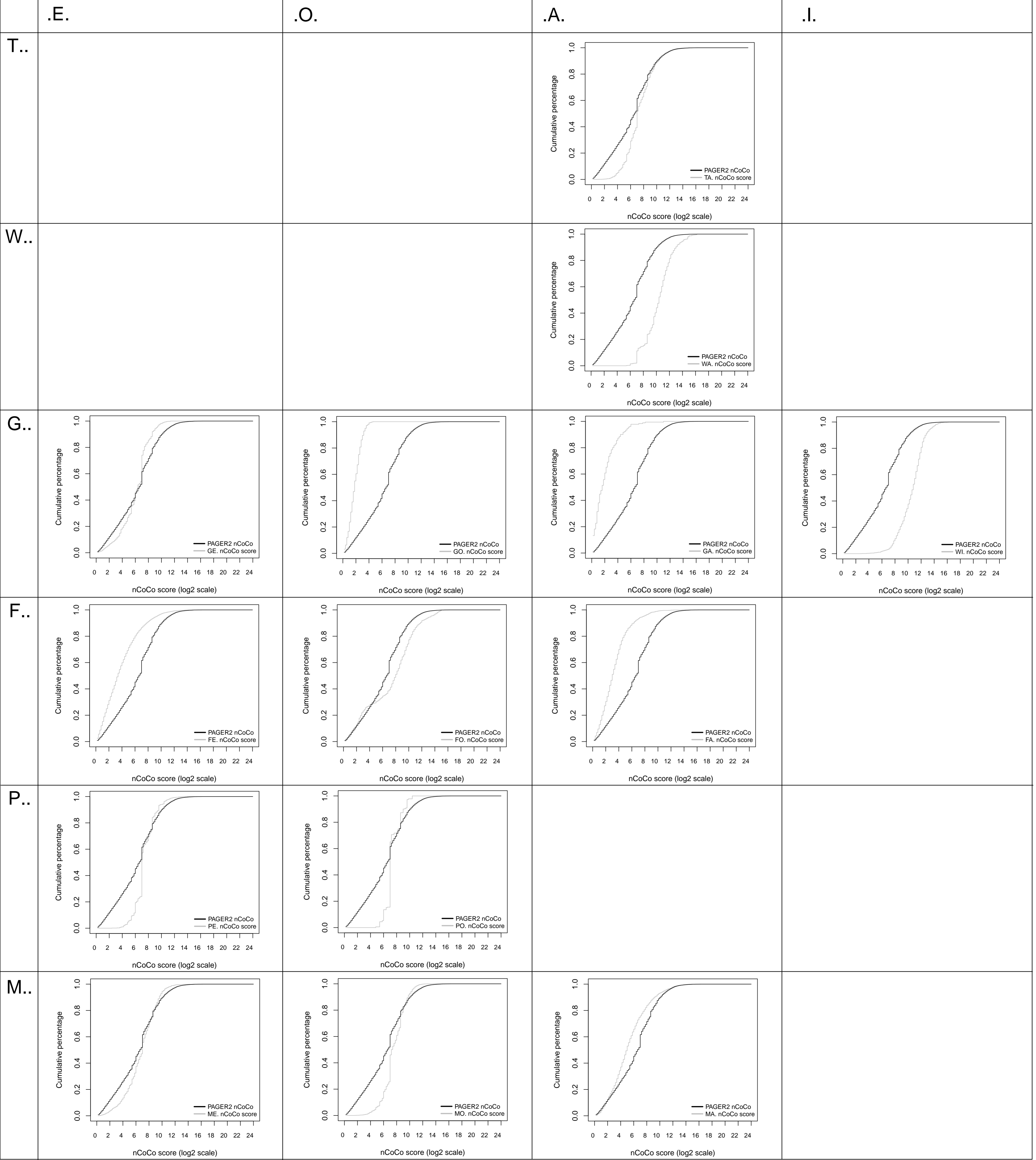

Supplement: Supplementary Data [file gkx1040_supp.zip › nar-02489-data-e-2017-File011.pdf]

**a**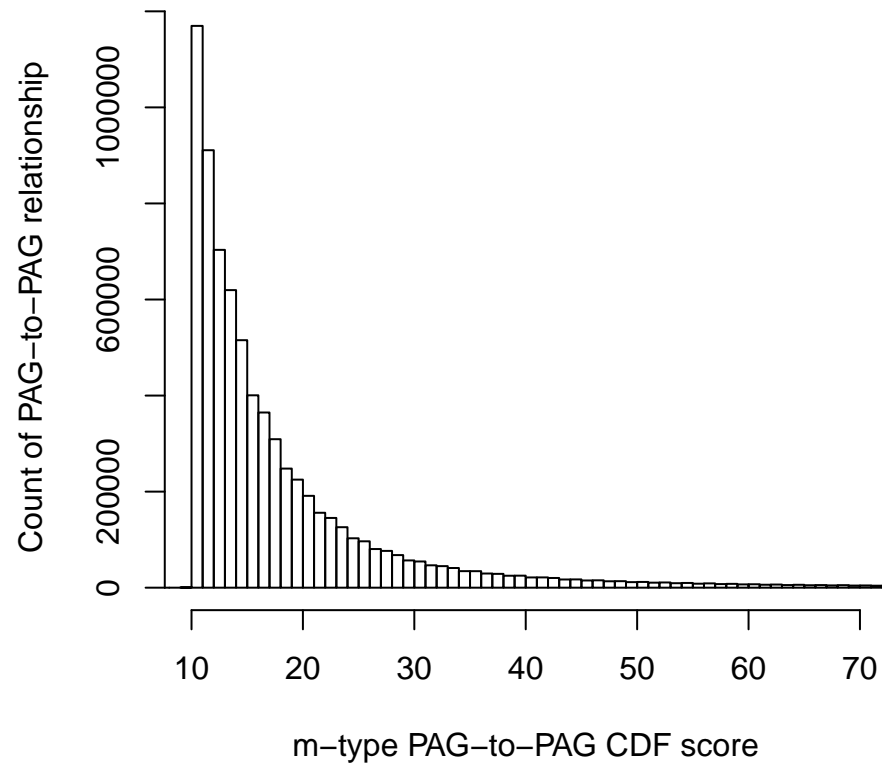**b**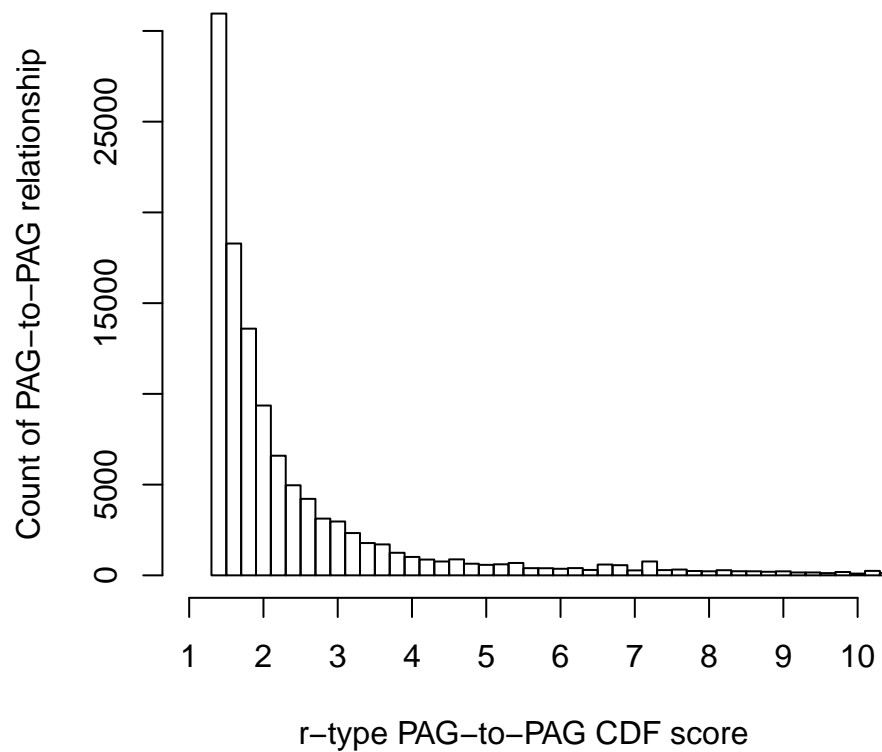

Supplement: Supplementary Data [file gkx1040_supp.zip › nar-02489-data-e-2017-File012.pdf]
